# Shear wave elastography to evaluate the effect of serum uric acid levels on carotid artery elasticity in patients with high-normal blood pressure

**DOI:** 10.5937/jomb0-52077

**Published:** 2025-03-21

**Authors:** Yifan Wang, Xinyu Wang, Xiya Li, Zhen Li, Mengmeng Zhang, Siran Zhang, Le Ma, Dongmei Huang, Guangsen Li

**Affiliations:** 1 The Second Affiliated Hospital of Dalian Medical University, Department of Ultrasound, Dalian, China

**Keywords:** shear wave elastography, high-normal blood pressure, carotid artery elasticity, serum uric acid level, elastografija smičnim talasom, visok normalan krvni pritisak, elastičnost karotidne arterije, nivo mokraćne kiseline u serumu

## Abstract

**Background:**

Populations with normal high blood pressure often already have elevated blood uric acid levels and a higher chance of developing atherosclerosis, which accelerates the process of cardiovascular disease. The risk of atherosclerosis increases with thickening of the carotid artery intima-media, and changes in carotid artery elasticity precede intima-media changes, so early monitoring of carotid artery elasticity is of great significance in the prevention of cardiovascular disease. Using shear wave elastography (SWE) to evaluate the effect of serum uric acid (SUA) levels on carotid artery elasticity in patients with high-normal blood pressure (BP).

**Methods:**

One hundred and fifteen patients with high-normal BP were selected, and then divided into tertiles according to the SUA levels. The left carotid intima-media thickness (IMT), peak systolic velocity (PSV) and diameter of common carotid artery were measured by two-dimensional ultrasound and Doppler flow imaging. Longitudinal elasticity of the left anterior carotid wall was measured by SWE, including the mean values of the minimum elastic modulus (MEmin), maximum elastic modulus (MEmax) and mean elastic modulus (MEmean).

**Results:**

IMT, MEmean, MEmin and MEmax were obviously higher in the 3rd tertile (all P < 0.05), while there were no obviously different between the 1st tertile and 2nd tertile. Pearson correlation analysis showed positive correlations between SUA and SWE-related parameters, while there was no correlation with IMT. Multiple linear regression analysis found that age, systolic BP, and SUA levels were independently associated with SWE-related parameters.

**Conclusions:**

SWE will be more helpful than IMT in monitoring the effect of blood uric acid levels on carotid artery elasticity in patients with high-normal BP.

## Abstract (sr)

**Uvod:**

Populacije sa normalnim visokim krvnim pritiskom često već imaju povišen nivo mokraćne kiseline u krvi i veće šanse za razvoj ateroskleroze, što ubrzava proces kardiovaskularnih bolesti. Rizik od ateroskleroze raste sa zadeblja njem intimamedija karotidne arterije, a promene elastičnosti karotidne arterije prethode promenama intimamedija, pa je rano praćenje elastičnosti karotidne arterije od velikog značaja u prevenciji kardiovaskularnih bolesti. Korišćenje elastografije smičnog talasa (SVE) za procenu uticaja nivoa mokraćne kiseline u serumu (SUA) na elastičnost karotidne arterije kod pacijenata sa visokim normalnim krvnim pritiskom (BP).

**Metode:**

Odabrano je 115 pacijenata sa visokim normalnim krvnim pritiskom, a zatim podeljeni na tertile prema nivoima SUA. Dvodimenzionalnim ultrazvukom i Doplerovim snimanjem mereni su debljina intime-medija leve karotide (IMT), vršna sistolna brzina (PSV) i prečnik zajedničke karotidne arterije. Uzdužna elastičnost levog prednjeg karotidnog zida merena je SVE, uključujući srednje vrednosti minimalnog modula elastičnosti (MEmin), maksimalnog modula elastičnosti (MEmak) i srednjeg modula elastičnosti (MEmean).

**Rezultati:**

IMT, MEmean, MEmin i MEmak su očigledno bili viši u 3. tertilu (svi P < 0,05), dok nije bilo očigledne razlike izme|u 1. tertila i 2. tertila. Pirsonova korelaciona analiza je pokazala pozitivne korelacije između parametara vezanih za SUA i SVE, dok sa IMT nije bilo korelacije. Višestruka linearna regresiona analiza je otkrila da su starost, sistolni krvni pritisak i nivoi SUA nezavisno povezani sa parametrima vezanim za SVE.

**Zaključak:**

SVE će biti od veće pomoći od IMT-a u praćenju uticaja nivoa mokra}ne kiseline u krvi na elastičnost karotidne arterije kod pacijenata sa visokim normalnim krvnim pritiskom.

## Introduction

Hypertension is a chronic disease that seriously endangers human health. A large survey in China showed that the morbidity of hypertension is increasing annually and the percentage of individuals with high-normal blood pressure (BP) is about 41.3% [1]. Many studies have demonstrated a link between serum uric acid (SUA) levels and high-normal BP, and administration of uric acid-lowering drugs such as allopurinol and probenecid could simultaneously reduce BP values [2] [3] [4]. Additionally, SUA can promote the formation of atherosclerosis (AS) by activating the renin-angiotensin-aldosterone system (RAAS), oxidizing low-density lipoproteins, and inducing the creation of reactive oxygen specie. And its injury to the blood vessels is more pronounced in patients with elevated BP [5]. However, owing to the influence of many confounding factors, the association between SUA levels and arterial stiffness appears to be inconsistent in different populations [6] [7]. The carotid intima-media thickness (IMT) is a conventional and reliable indicator for the assessment of AS. But generally, endothelial dysfunction precedes structural changes. Shear wave elastography (SWE) is a novel ultrasound technology that can calculate the absolute value of Young's modulus by measuring the propagation velocity of the shear wave through the tissue, thus providing a quantitative assessment of the tissue hardness [8]. Several studies have identified that SWE can be applied to the detection of artery elasticity [9] [10]. We aimed to assess the effect of different SUA levels on carotid artery elasticity in patients with highnormal BP by using SWE technology and to investigate the independent influencing factors of carotid artery elastic modulus in this population.

## Materials and methods

### Study population

A total of 115 patients with high-normal BP without known history of cardiovascular diseases in our hospital from August 2021 to May 2023 were recruited in this research. All participants met the global hypertension practice guidelines published by the International Society of Hypertension in 2020 [11]. According to the SUA tertiles, patients with high-normal BP were classified into three groups: 1st tertile (38 patients, SUA < 4.23 mg/dL, mean age: 36.87±6.60 years); 2nd tertile (38 patients, 4.23 mg/dL SUA 5.56 mg/dL, mean age: 36.82±8.07 years); 3rd tertile (39 patients, SUA > 5.56 mg/dL, mean age: 37.10±7.02 years). Exclusion criteria included: 1) history of hypertension or treatment with anti-hypertensive drugs. 2) stroke, myocardial infarction, peripheral vascular diseases, and heart failure. 2) diabetes mellitus, severe liver or kidney insufficiency. 3) infection, tumor, or immune system deficiency. 4) hyper-or hypothyroidism. 5) use of uric acid-lowering drugs or hormone drugs. 6) Recent oral or respiratory tract inflammation resulting in the presence of visibly enlarged lymph nodes in the neck. In addition, patients with respiratory hyperactivity and excessive carotid artery pulsation that made the elastography unstable were also excluded. All subjects had no carotid plaque and IMT 1.0 mm.

### Clinical and biochemical data

Basic information and clinical data of all subjects including age, height, weight, BP, and heart rate were recorded and then we calculated body mass index (BMI). We also collected biochemical parameters such as SUA, triglycerides (TG), fasting blood glucose (FBG), high-density lipoprotein (HDL-C), total cholesterol (TC), and low-density lipoprotein (LDL-C).

### Carotid ultrasonography

All subjects were supine on the examination bed in a calm state with the head tilted back and to the opposite side to fully expose the carotid sweep area. Conventional carotid ultrasonography was performed using a Mindray diagnostic ultrasound machine (Resona 8; Mindray, Guangzhou province, China) equipped with an L14-5 WU linear probe (frequency of 10-12 MHz). After adjustment to the long-axis section of the carotid artery, the artery was kept flat and the conventional parameters were measured 1-1.5 cm below the left common carotid bifurcation. The diameter and peak systolic flow velocity (PSV) of the common carotid artery were obtained by twodimensional ultrasound and Doppler flow imaging.

### SWE measurements

**Figure 1:**
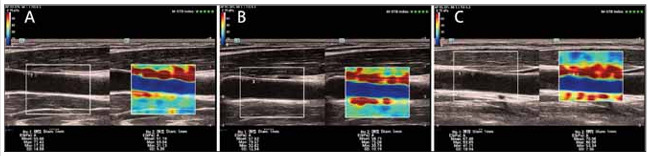
SWE-related parameters of the participants stratified by SUA tertiles. The SUA levels were divided into tertiles including (A) group A (SUA level<4.23 mg/dL), (B) group B (SUA level 4.23–5.56 mg/dL), and (C) group C (SUA level>5.56 mg/dL).

The ultrasonic probe was placed on the surface of the neck skin. We adjusted the depth and gain until the two-dimensional images were clearly displayed, and then we switched to SWE mode. All subjects were required to hold their breath and instructed not to swallow during the scan. The diameter of the circular sampling frame was set to 1 mm. The measurements were taken at 2 mm intervals along the carotid intima and then averaged. We repeated the measurements three times and then averaged to obtain MEmax, MEmin and MEmean (Figure 1).

Thirty subjects were randomly selected from the cohort to assess intra-and inter-observer variability for SWE-related parameters.

### Statistical analysis

Collected data were statistically analyzed using SPSS 25.0 software and MedCalc 20.1.0 software. Measured data conforming to normal distribution were expressed as mean ± SD. Statistical differences between the three groups were compared using oneway ANOVA and LSD-t test. Pearson linear correlation analysis was used to analyze the correlations between SUA levels and SWE-related parameters and IMT. Multiple linear regression analysis was performed to analyze independent risk factors for SWE-related parameters. The intra-and inter-observer variability were quantified by the Bland-Altman plots. *P* < 0.05 indicated significant differences.

## Results

### General information

TG increased progressively with the increasing SUA tertiles, whereas HDL-C decreased (all *P* < 0.05) (Table 1). BMI, LDL-C, IMT were higher in the 3rd tertile (all *P* < 0.05), while there was no difference between the 1st tertile and 2nd tertile (all *P* > 0.05) (Table 2). 

**Table 1:** Clinical characteristics of the study population according to SUA tertiles. Abbreviations: SUA, serum uric acid; SBP, systolic blood pressure; DBP, diastolic blood pressure; BMI, body mass index; TC, total cholesterol; TG, triglycerides; HDL-C, high-density lipoprotein-cholesterol; LDL-C, low-density lipoprotein-cholesterol; FBG, fasting blood glucose. I:1st tertile; II:2nd tertile; III:3rd tertile.<br>^*^ *P*< 0.05 vs group I. ^#^ *P*< 0.05 vs group II. Data are expressed as n (%), or as mean ± standard deviation.

Variables	I (n=38)	II (n=38)	III (n=39)	*P* value
SUA level (mg/dL)	<4.23	4.23~5.56	>5.56	0.000
Age (y)	36.87±6.60	36.82±8.07	37.10±7.02	0.983
Heart rate (bpm)	72.37±7.59	73.58±7.36	70.23±9.00	0.184
SBP (mmHg)	131.03±4.37	130.97±5.13	132.62±4.12	0.202
DBP (mmHg)	83.42±3.89	84.63±3.11	84.82±2.70	0.132
BMI (kg/m^2^)	23.34±2.77	23.89±3.52	25.59±3.97^*#^	0.014
TC (mmol/L)	4.83±0.77	4.92±0.78	5.14±0.65	0.168
TG (mmol/L)	1.15±0.42	1.40±0.44^*^	1.69±0.59^*#^	0.000
HDL-C (mmol/L)	1.44±0.29	1.30±0.30^*^	1.14±0.23^*#^	0.000
LDL-C (mmol/L)	3.10±0.73	3.34±0.72	3.56±0.81^*^	0.035
FBG (mmol/L)	4.83±0.60	4.93±0.53	4.99±0.57	0.445

**Table 2:** Carotid artery parameters of the study population according to SUA tertiles. Abbreviations: CCA, common carotid artery; PSV, peak systolic velocity.<br>^*^ *P* < 0.05 vs group I. ^#^ *P* < 0.05 vs group II. Data are expressed as n (%), or as mean ± standard deviation.

Variables	I (n=38)	II (n=38)	III (n=39)	*P* value
IMT (mm)	0.55±0.07	0.58±0.09	0.63±0.10^*#^	0.001
Diameter of CCA (mm)	6.90±0.32	7.03±0.42	6.97±0.36	0.314
PSV (cm/s)	74.61±13.67	71.80±14.02	75.42±12.51	0.466
MEmean (kPa)	48.71±12.58	52.69±13.88	59.39±17.28^*#^	0.007
MEmax (kPa)	58.24±14.64	62.64±16.31	72.04±18.00^*# ^	0.001
MEmin (kPa)	39.12±12.02	42.69±13.70	49.14±16.02^*#^	0.008

### Results of carotid artery elasticity parameters

MEmean, MEmin and MEmax were higher in the 3rd tertile (all *P* < 0.05), while there was no difference between the 1st tertile and 2nd tertile (all *P* > 0.05) (Table 2).

### Results of correlation analysis

**Figure 2:**
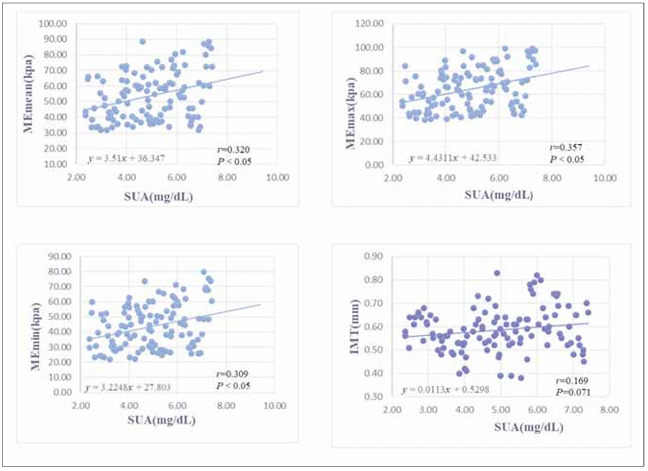
The correlation of serum SUA level with MEmax, MEmean, MEmin and IMT in high-normal BP patients by scatter plot.

Correlation analysis confirmed that MEmax, MEmean and MEmin were positively correlated with SUA (r=0.320, 0.357, 0.309, *P*<0.05). While, no correlation was found between IMT and SUA (r=0.169, *P*>0.05) (Figure 2). Multiple linear regression analysis showed that age, systolic BP, and SUA levels were independently correlated with SWE-related parameters (Table 3).

**Table 3:** Relation between conventional risk factors and SWE-related parameters as determined by linear regression analysis. Abbreviations: BMI, body mass index; SBP, systolic blood pressure; HDL-C, high-density lipoprotein-cholesterol; SUA, serum uric acid. *P*< 0.05 are indicated in bold. Data are expressed as n (%), or as mean ± standard deviation.

	MEmax		MEmean		MEmin	
	β	*P* value	β	*P* value	β	*P* value
Age	0.210	0.016	0.197	0.028	0.197	0.028
BMI	-0.041	0.637	-0.027	0.761	-0.010	0.906
SBP	0.238	0.009	0.234	0.012	0.230	0.013
Heart rate	-0.026	0.752	0.011	0.897	0.016	0.854
HDL-L	-0.149	0.115	-0.140	0.149	-0.174	0.074
SUA	0.236	0.018	0.204	0.045	0.175	0.086

### Reproducibility test results

**Figure 3:**
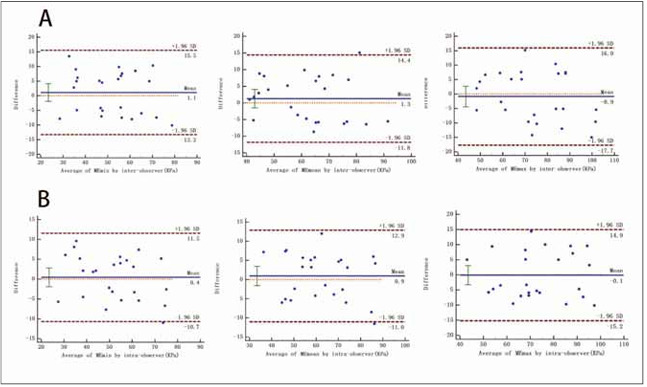
Repeatability was analyzed by Bland-Altman Plots. (A), Bland–Altman plot of interobserver variability. (B), Bland–Altman plot of intraobserver variability.

Intra and inter-observer reliabilities for MEmean, MEmax, and MEmin were high (Figure 3).

## Discussion

High-normal BP is considered as an intermediate state between normotension and hypertension. According to the U.S. National Health and Nutrition Examination Survey, the incidence of high-normal BP decreases with age, unlike the incidence of hypertension [12]. It is indicated that a considerable portion of patients with high-normal BP may gradually progress to hypertension, further aggravating the occurrence of cardiovascular complications. As a highly heterogeneous disease, the progression of hypertension is influenced by many factors. Tatsumi et al. [13] retrospectively analyzed the clinical data of 7848 participants who underwent a physical examination. They found that after adjusting for the effects of alcohol consumption, hyperuricemia is still an independent risk factor associated with hypertension. Urid acid, the end product of purine metabolism in the body, can cause cardiovascular damage when it is abnormally high [14]. Research has demonstrated that patients with high SUA levels had a 2.5-fold higher risk of death from cardiovascular disease (CVD) than those with low SUA levels [15]. In addition, other studies have found that each 1mg/dL increase in SUA exhibited a 32% increase in the incidence of CVD, equivalent to each 46 mg/dL increase in cholesterol or each 10mmHg increase in systolic BP [16].

AS is the earliest recognized event in the development of most CVDs [17]. Some high-risk factors can cause systemic endothelial injury, resulting in a range of pathophysiological responses including lipoprotein oxidation, endothelial dysfunction, free oxygen radical production, and cell migration [18]. Owing to the superficial location and the similarity of the vessel wall structure and flow environment to the coronary arteries, the carotid artery is commonly used as a window for monitoring systemic large and medium arterial sclerosis. Conventional ultrasound assesses arterial elasticity by measuring IMT and has the advantages of being non-invasive, simple, and reproducible [19]. However, IMT is a morphological indicator of pathological arterial wall thickening, and its abnormalities often appear later than those of arterial function. SWE is a novel ultrasound technology for examining the stiffness of living tissues [20] [21]. The rationale is that the probe emits safe pulses of acoustic radiation that generates transverse shear waves by focusing continuously on different tissue depths to obtain the real-time elastography [22]. Unlike other techniques for assessing arterial elasticity, SWE can characterize the viscoelasticity of the arterial wall at a fine level in time and space [23]. Therefore, more information on arterial wall heterogeneity may be generated. Wang et al. [10] used the SWE technique to measure carotid artery elasticity in patients with coronary slow flow. They confirmed that it is a simple and effective method that can contribute to the prevention and monitoring of cardiovascular events and adverse outcomes. Additionally, a systematic review has shown that SWE is more reliable than the results obtained by conventional ultrasound and shows good to excellent reproducibility in assessing arterial elasticity [9].

In our study, IMT, MEmean, MEmin, and MEmax were higher in the 3rd tertile compared with the 1st tertile and 2nd tertile. This means that in patients with high-normal BP, carotid artery elasticity gradually decreases with increasing SUA levels. The reason for this may be that the increased blood flow is constantly impacting the arterial wall, causing damage or even rupture of the walls. Moreover, SUA has very low physical solubility in the blood and tends to form uric acid crystals and deposits on the arterial wall. The deposited uric acid crystals will directly damage the artery intima and cause inflammatory response in the intima [24]. Therefore, in a relatively poor artery condition, uric acid crystals may be more likely to cause a decrease in the artery wall elasticity and promote the development of AS. In addition, SUA can also indirectly affect arterial elasticity by activating the RAAS and the sympathetic nervous system, oxidizing low-density lipoproteins, and activating inducible nitric oxide synthase to produce cytotoxicity [25] [26]. The damage to blood vessels from SUA will advance the development of hypertension, thereby creating a vicious cycle. Cipolli et al. [27]. found no correlation between IMT and SUA in the subgroup with elevated SUA as well as in the subgroup with normal SUA, no matter in female or male. In addition, a study from Malaysia recruited 245 patients with longterm follow-up hypertension. All patients had BP control close to the high-normal BP category. They also found no significant correlation between IMT and SUA under conditions of good BP control [28]. Similar to the above-mentioned study, we found that SUA was positively correlated with MEmean, MEmin, and MEmax, but not with IMT in patients with highnormal BP, suggesting that SUA may primarily affect the elastic function of the artery rather than the arterial structure. Different results have been previously observed in patients with hypertension. In this study, we found that age, systolic BP, and SUA levels were independently associated with SWE-related parameters. And as an independent risk factor, SUA levels seemed to have a small potential effect on arterial elastic modulus but remained significant. This contradicted to a part of published findings, probably because in our study only untreated patients were included in the analysis and drug treatment was known to have a strong effect on arterial elasticity. Secondly, our participants were relatively young and BP values were between the normal and hypertensive categories, thus not masking the effects of secondary factors.

In addition to shear wave elastography, conventional ultrasound also has a number of imaging techniques for assessing carotid artery elasticity. Compared with traditional ultrasound imaging techniques for arterial elasticity assessment, SWE requires only the assistance of the ultrasound probe and does not require the user to apply external pressure at all for objective and quantitative assessment of tissue elasticity [29]. This avoids the limitations of the traditional external pressure ultrasound elasticity imaging techniques such as the influence of subjective experience, the lack of objective and quantitative indexes, and the application range of superficial tissues only. In recent years, a commonly used and reliable ultrasound technique to assess elasticity is pulse wave velocity (PWV), which has many advantages, but due to technical limitations, PWV can only be applied to superficial carotid arteries at present. However, it has higher requirements for heart rate and breath holding, which is not operable for some elderly patients [30]. Therefore, SWE has more advantages than PWV in assessing carotid artery elasticity.

There are several limitations of our study. Firstly, the impact of sample size and single-center studies may limit generalization and extrapolation. Secondly, there are difficulties in acquiring images of SWE for those patients with respiratory hyperactivity and excessive carotid artery pulsation.

## Conclusion

In conclusion, SWE can effectively and noninvasively assess the effect of SUA levels on carotid artery elasticity in patients with high-normal BP. Aggressive clinical management of such patients before irreversible cardiovascular damage is extremely important to improve patient prognosis. And in this area, SWE may help provide valuable clinical evidence.

## Dodatak

### Conflict of interest statement

All the authors declare that they have no conflict of interest in this work.

### Contribution

Yifan Wang and Xinyu Wang contributed equally to this work.
